# Urinary Metabolites of Organophosphate and Pyrethroid Pesticides and Behavioral Problems in Canadian Children

**DOI:** 10.1289/ehp.1306667

**Published:** 2013-10-22

**Authors:** Youssef Oulhote, Maryse F. Bouchard

**Affiliations:** 1 Department of Environmental and Occupational Health, Université de Montréal, Québec, Canada; 2 CHU (Le centre hospitalier universitaire mère-enfant) Sainte-Justine, Montréal, Québec, Canada

## Abstract

Background: Exposure to organophosphate pesticides has been associated with neurobehavioral deficits in children, although data on low levels of exposure experienced by the general population are sparse. Pyrethroids are insecticides rapidly gaining popularity, and epidemiological evidence on their potential effects is lacking.

Objective: We examined the association between exposure to organophosphate and pyrethroid pesticides, indicated by urinary metabolites, and parentally reported behavioral problems in children.

Methods: We used data on children 6–11 years of age from the Canadian Health Measures Survey (2007–2009). We used logistic regressions to estimate odds ratios (ORs) for high scores on the Strengths and Difficulties Questionnaire (SDQ), which may indicate behavioral problems, in association with concentrations of pyrethroid and organophosphate metabolites in the urine of 779 children, adjusting for covariates (sex, age, race/ethnicity, income, parental education, blood lead levels, maternal smoking during pregnancy, and others).

Results: At least one urinary metabolite for organophosphates was detected in 91% of children, and for pyrethroids in 97% of children. Organophosphate metabolites were not significantly associated with high SDQ scores. The pyrethroid metabolite *cis*-DCCA [3-(2,2-dichlorovinyl)-2,2-dimethylycyclopropane carboxylic acid] was significantly associated with high scores for total difficulties on the SDQ (OR for a 10-fold increase = 2.0; 95% CI: 1.1, 3.6), and there was a nonsignificant association with *trans*-DCCA (OR = 1.6; 95% CI: 0.9, 3.0).

Conclusion: In contrast with previous studies, we did not observe an association between exposure to organophosphate pesticides and behavioral scores in children. However, some pyrethroid urinary metabolites were associated with a high level of parent-reported behavioral problems. Longitudinal studies should be conducted on the potential risks of pyrethroids.

Citation: Oulhote Y, Bouchard MF. 2013. Urinary metabolites of organophosphate and pyrethroid pesticides and behavioral problems in Canadian children. Environ Health Perspect 121:1378–1384; http://dx.doi.org/10.1289/ehp.1306667

## Introduction

Organophosphate and pyrethroid compounds are the most widely used pesticides worldwide. Organophosphates are used in Canada and throughout the world for crop protection, control of vector-borne diseases, and residential pest control (Sudakin and Power 2007). Although the quantity of organophosphate pesticides is slowly decreasing because of concern for toxic effects, the use of pyrethroids is rapidly increasing. Pyrethrins and their synthetic derivatives, pyrethroids, have become the predominant insecticide class for control of vector-borne diseases and residential uses such as pest infestation, fleas and ticks on pets, and head lice (Walters et al. 2009). In addition, pyrethroid insecticides are used for insect control on agricultural crops (Agency for Toxic Substances and Disease Registry 2003).

Organophosphate and pyrethroid pesticides interfere with the function of the nervous system of insects, and to a lesser degree with that of mammals, thus raising concerns for human health. Residue on foods is the primary exposure source to organophosphate pesticides (Lu et al. 2008), and both residues on foods and household pest control products are important exposure sources for pyrethroids (Morgan 2012). Inhalation, dermal exposure, and nonintentional ingestion also may be important exposure routes for pesticides (Bradman and Whyatt 2005). Children are at greatest risk from pesticide toxicity because the developing brain is more susceptible to neurotoxicants, and they interact with their environment in particular ways such as frequent hand-to-mouth behavior and outside play (Roberts et al. 2012). In addition, children have higher levels of internal exposure than adults for a given level of environmental exposure because of lower body weight, and because their enzymatic detoxification mechanism for organophosphate pesticides is not fully developed (Huen et al. 2009).

Prenatal exposure to organophosphate pesticides has been associated with increased psychomotor and mental development delays, attention problems, attention deficit/hyperactivity disorder (ADHD)–like problems, and symptoms consistent with pervasive developmental disorder (Eskenazi et al. 2007; Marks et al. 2010; Rauh et al. 2006) and negatively associated with IQ scores (Bouchard et al. 2011). In addition, postnatal exposure to organophosphate pesticides has been associated with parent-reported problems with motor skills and behavior, poorer short-term memory and attention (Ruckart et al. 2004), slower motor speed (Rohlman et al. 2005), longer reaction time (Grandjean et al. 2006), and ADHD in children (Bouchard et al. 2010).

Despite common use of pyrethroids, potential risks of low levels of exposure to pyrethroids in children are poorly understood. To our knowledge, the only epidemiological study published on this topic—a study of 348 mother–child pairs in New York City—assessed prenatal exposure to pyrethroids based on personal air sample measurements during pregnancy, but did not evaluate postnatal exposure (Horton et al. 2011). The authors reported that prenatal exposure to piperonyl butoxide—a synergist for pyrethroid insecticides—was associated with significantly lower Bayley Scales of Infant Development (BSID) Mental Development Index scores at 3 years of age (Horton et al. 2011). Low-level exposure to some common pyrethroid insecticides has been shown to negatively affect the development of nervous system in young laboratory animals, manifested in persistent changes in learning, motor activity, and sexual behavior (Lazarini et al. 2001; Moniz et al. 1994).

Few studies have investigated neurobehavioral outcomes associated with exposures to pesticides at levels experienced by children in the general population. Here, we report on associations between exposures to organophosphate and pyrethroid pesticides, indicated by urinary metabolites, and parentally reported evidence of behavioral problems in Canadian children at 6–11 years of age.

## Material and Methods

*Survey design and participants*. We used data from the Canadian Health Measures Survey (CHMS; cycle 1, 2007–2009), a complex-design survey that uses multistage probability sampling and oversampling of certain subgroups to generate a study sample that is representative of the general Canadian population (Statistics Canada 2011). Participants completed a household survey that included questions about demographics and health history, and provided blood and urine samples during physical examinations at the mobile examination center. For cycle 1 of CHMS, data were collected from approximately 5,600 Canadians 6–79 years of age, including 1,081 children 6–11 years of age, in 15 sites spread across Canada. We limited our analysis to these children because information on early-life factors important for neurodevelopment was available only for children in this age range. The CHMS was approved by the Health Canada institutional review board; all adult participants provided written informed consent, and all children gave their assent to participate.

*Assessment of children’s behavioral problems*. Behavioral problems were assessed using the parent version of the Strengths and Difficulties Questionnaire (SDQ) (Goodman 1997). The SDQ is an internationally used and validated screening questionnaire designed to assess the mental and behavioral difficulties and strengths of 3- to 16-year-old children (Goodman 2001). A validity study was conducted on a large sample (*n* = 18,415) representative of children and adolescents 11–16 years of age living in Great Britain (Goodman and Goodman 2009). The findings showed that children with higher scores on the SDQ (total difficulties score) had a higher prevalence of clinical psychopathology disorders, based on a multi-informant–based clinician-rated clinical diagnosis. Higher SDQ scores also predicted clinical diagnoses in a subset of children (*n* = 7,912) that completed a 3-year follow-up evaluation.

The SDQ is intended for use in population surveys, and is acceptable to parents. The questionnaire is composed of 25 items scored on a 3-point scale: 0 (“not true”), 1 (“somewhat true”), and 2 (“certainly true”). A global total difficulties score is calculated, in addition to scores for five dimension scales: emotional symptoms, conduct problems, hyperactivity/inattention, peer problems, and prosocial behavior. Scores for total difficulties, which are calculated by summing the scores of 4 of the 5 dimension scales (all but prosocial behavior), range from 0 to 40, whereas scores on the dimension scales range from 0 to 10. Scores can be classified as normal, borderline, or high. As recommended by the author of the instrument, in the present study we grouped scores into two categories: high versus low/borderline, using these cut-off scores: emotional symptoms ≥ 5, conduct problems ≥ 4, hyperactivity/inattention ≥ 7, peer problems ≥ 4, prosocial behavior ≤ 4, and total difficulties ≥ 17 (Goodman 2001).

*Measurements of pesticides metabolites*. A spot urine sample was collected from each child within 2 weeks of survey questionnaire completion (20-mL samples), and kept frozen for storage at –20°C until analysis at the Institut National de Santé Publique du Québec. In addition, urinary creatinine concentration was measured to account for urine dilution. Six urinary dialkyl phosphate (DAP) metabolites that result from the degradation of different organophosphates were measured in urine, and concentrations were summed to provide an indicator of total exposure to this class of pesticides (National Research Council 1993). The urinary DAP metabolites measured are three dimethyl alkylphosphate (DMAP) molecules [dimethylphosphate (DMP), dimethylthiophosphate (DMTP), dimethyldithiophosphate (DMDTP)] and three diethyl alkylphosphate (DEAP) molecules [diethylphosphate (DEP), diethylthiophosphate (DETP), diethyldithiophosphate (DEDTP)].

For pyrethroids, five urinary metabolites were measured: 4-fluoro-3-phenoxybenzoic acid (4-F-3-PBA), *cis*-3-(2,2-dibromovinyl)-2,2-dimethylcyclopropane-1-carboxylic acid (*cis*-DBCA), *cis*-3-(2,2-dichlorovinyl)-2,2-dimethylcyclopropane carboxylic acid (*cis*-DCCA), *trans*-3-(2,2-dichlorovinyl)-2,2-dimethylcyclopropane carboxylic acid (*trans*-DCCA), and 3-phenoxybenzoic acid (3-PBA). The laboratory methods can be found elsewhere [Institut national de santé publique du Québec (INSPQ) 2009a, 2009b]. Similar to organophosphates, the metabolic pathways for different parent compounds produce the same breakdown products. The *cis-* and *trans-*DCCA are metabolites of the *cis-* and *trans-*isomers of cypermethrin, cyfluthrin, or permethrin, and 3-PBA is a metabolite common to several pyrethroids. Metabolites of organophosphate and synthetic pyrethroids in urine primarily reflect recent exposure to the parent compounds because they are quickly metabolized and excreted, with half-lives of 2 hr to a few days (Kaneko and Miyamoto 2001; Wessels et al. 2003).

*Questionnaire data on domestic use of pesticides*. Each parent was asked three questions about pesticide use in the preceding month, specifically, chemicals used indoors to control roaches, ants, termites, or other insects; chemicals used to control head lice on family members or fleas on pets; and chemicals used on the lawn, yard, or the surrounding fields, woods, or orchards to kill insects or weeds, or to control plant diseases.

*Covariates and possible confounders*. We included blood lead levels (as a continuous value) and maternal smoking during pregnancy (parent report of smoking regularly during any stage of pregnancy) *a priori* in models because they are important risk factors for behavioral problems in children (Braun et al. 2006, 2008). We examined the following variables as potential confounders: sex, age (years), race/ethnicity (white/nonwhite; there were very few nonwhite children, so we grouped individuals from all other cultural and ethnic origins in the nonwhite category, including Hispanics), family income (tertiles: 0–59,999; 60,000–99,999; ≥ 100,000 Can$), parental education (with/without high school diploma), birth weight (grams), maternal age at child’s birth (years), current exposure to environmental tobacco smoke (parent report of daily tobacco smoking inside the home), child’s body mass index (BMI; as a continuous value), and fasting status (fasting duration at urine collection > 10 hr/≤ 10 hr). Each variable was examined in a bivariate analysis for its association with high SDQ total difficulties score, and with concentrations of urinary pesticides metabolites (logarithmically transformed). Variables that predicted both the total difficulties score and the exposure with *p* < 0.2 (chi-square test, one-way analysis of variance, or Spearman correlation depending on whether variables were categorical or continuous) were retained as model covariates. In addition, we included urinary creatinine concentration as a covariate in models to account for urine dilution. We chose this approach rather than standardizing urinary concentrations for creatinine to reduce potential bias that may be introduced by age-, race/ethnicity-, and sex-related variation in creatinine excretion (Barr et al. 2005).

*Statistical analyses*. We used logistic regression to estimate associations between urinary pesticide metabolite concentrations and high scores on the SDQ for total difficulties, and on four of the five dimension scales (too few children had high scores on the fifth dimension scale—prosocial behavior—to compute estimates of association). Models used to estimate associations with the pyrethroid metabolites *cis*- and *trans*-DCCA included sex, age, race/ethnicity, income, parental education, blood lead levels, maternal smoking during pregnancy, birth weight, and urinary creatinine. Models used to estimate associations with the pyrethroid metabolite 3-PBA or with ΣDAP also included BMI and fasting status. Covariates were entered in the model with the categories described in “Covariates and possible confounders.”

We log-transformed (base 10) urinary concentrations of pesticide metabolites, creatinine, and blood lead to normalize their distributions. Separate models were used to estimate associations with the urinary pyrethroid metabolites *cis*-DCCA, *trans*-DCCA, and 3-PBA. We did not estimate associations with the metabolites *cis*-DBCA and 4-F-3-PBA because > 50% of children had urinary levels below the limit of detection (LOD) (Table 1). The three other pyrethroid metabolite levels were < LOD in < 3% of samples, so we imputed them using regression on order statistics (Helsel 2005), a method that uses quantile regression to predict concentrations < LOD based on concentrations > LOD. This is a robust and less biased procedure than the maximum likelihood estimation under the log-normal distribution (Shumway et al. 2002). Urinary DAP concentrations were divided by their respective molecular weights before being summed to yield ΣDEAPs, ΣDMAPs, and total ΣDAPs concentrations (nanomoles per liter). For DAP metabolites, associations with the outcomes were estimated for summative measures (i.e., ΣDEAPs and ΣDMAPs) rather than for individual analytes. Therefore, we imputed values < LOD even for analytes with low detection frequencies, because the summative measures were much more influenced by concentrations with valid measurement than by imputed values. Finally, three creatinine values were missing; we imputed them with the value LOD/_√_^–^2.

**Table 1:** Concentrations of organophosphate and pyrethroid pesticides urinary metabolites for children 6–11 years of age.

Exposure	*n*	Detection limit	Percent < LOD	Median	IQR	Maximum
Organophosphate pesticide metabolites (nmol/L)
DMP	1,035	7.9	19.3	34.6	10.8–91.9	1,428
DMTP	1,036	4.2	32.5	17.6	< LOD–75.4	2,233
DMDTP	1,036	1.9	59.5	< LOD	< LOD–5.6	716
DEP	1,036	3.9	19.0	19.6	8.5–42.0	4,849
DETP	1,036	3.5	58.4	< LOD	< LOD–6.9	1,656
DEDTP	1,036	1.6	96.7	< LOD	< LOD–< LOD	40
∑DMAP	1,035	NA	15.4^*a*^	62.0	18.7–192.8	2,905
∑DEAP	1,036	NA	18.5^*a*^	25.0	10.5–51.3	4,849
∑DAP	1,035	NA	8.4^*a*^	99.2	34.3–273.3	4,877
Pyrethroid pesticide metabolites (μg/L)
*cis*-DBCA	981	0.006	52.0	< LOD	< LOD–0.02	2
4-F-3-PBA	1,005	0.008	58.7	< LOD	< LOD–0.01	3
3-PBA	1,032	0.01	0.7	0.20	0.10–0.42	32
*cis*-DCCA	1,033	0.007	2.7	0.05	0.03–0.10	6
*trans*-DCCA	1,034	0.01	0.1	0.15	0.08–0.35	41
Abbreviations: IQR, interquartile range; NA, not applicable. ^***a***^Percentage of participants with all values in the sum < LOD.						

In additional analyses, we explored possible sex-related differences in the association between urinary pesticide metabolites and behavioral problems by introducing a term for sex × urinary level in the models. We set the threshold for statistical significance at *p* < 0.1 for interactions. We also explored possible interactions between blood lead level (< 0.9/≥ 0.9 μg/dL) and pesticides, but all *p*-values were > 0.1. Finally, we conducted a complementary analysis to assess the association between SDQ scores and categories of pesticide use (indoor use, outdoor use, use to control head lice or insects on pets, and any use), adjusting for the same covariates as those used in the models for *cis*- and *trans*-DCCA.

We set the threshold for statistical significance at *p* < 0.05, and all tests were two-sided. We used Stata (version 11; StataCorp, College Station, TX, USA) for all analyses. To account for the complex sampling design, we estimated sample variances and 95% CIs using bootstrapped weights (*n* = 500) (Rust and Rao 1996). The bootstrap resampling method was used to account for the sampling design and to obtain variance estimates using the mean square error formula. As recommended by Statistics Canada (2011) for analysis of CHMS data, we restricted the number of degrees of freedom to 11 to account for the number of collection sites and sampling stratiﬁcation.

We performed a few sensitivity analyses. First, we repeated models adjusting for the ratio of pesticide metabolite to creatinine level, instead of adjusting for creatinine. Second, because 149 children with missing data for blood lead were excluded from models adjusted for lead, we ran the models without adjusting for blood lead levels, thus increasing the analytical sample to 905 children. To make the results comparable with the main analysis, we also ran models without lead adjustment on the 779 children in the main analysis. Third, we performed unweighted analyses that did not account for the survey design. This is important because the weighted method may result in an inefficient analysis due to the large variability in assigned weights, especially when there are few degrees of freedom, as in the CHMS. The unweighted analysis should yield correctly estimated odds ratios (ORs) when models are adjusted for the variables used to define the weights (i.e., age, sex, and ethnicity) (Korn and Graubard 1991).

## Results

*Description of the population and levels of urinary pesticide metabolites*. There were 1,081 children 6–11 years of age in the CHMS 2007–2009. Measures of urinary levels of most pesticide metabolites and behavioral assessment with the SDQ were available for 1,030 children. The median of ΣDAP metabolites in urine was 99.2 nmol/L (Table 1). ΣDMAP metabolite concentration (median, 62.0 nmol/L) was considerably higher than that of ΣDEAP metabolites (median, 25.0 nmol/L). The median concentrations for pyrethroid metabolites were 0.05 μg/L, 0.15 μg/L and 0.20 μg/L, respectively, for *cis*-DCCA, *trans*-DCCA, and 3-PBA. The median blood lead concentration was 0.9 μg /dL.

Table 2 presents the population characteristics. Children were predominantly white (75%) and had parents with relatively high education levels (80% with at least a high school degree). The percentage of parents reporting using chemicals in the month before the interview for controlling roaches, ants, termites, or other insects indoors was 3.8%, and 4.3% reported using chemicals to treat head lice on family members or fleas and ticks on pets. In addition, 7.3% reported that their lawn or yard, or the surrounding fields, woods, or orchards, had been treated with chemicals to kill insects or weeds or control plant diseases. Overall, 14% of the children lived in a household where pesticides had been used for any of those purposes in the previous month.

**Table 2:** Concentration of organophosphate (nmol/L) and pyrethroid (μg/L) urinary metabolites according to characteristics of study participants (weighted statistics; *n* = 1,030).

Participant characteristics	*n* (%)	GM (SE)			
		∑DAP	*cis*-DCCA	*trans*-DCCA	3-PBA
Age (years)
6–8	458 (47)	103.9 (8.2)	0.06 (0.01)	0.18 (0.02)	0.22 (0.03)
9–11	572 (53)	87.2 (8.4)	0.05 (0.01)	0.17 (0.02)	0.20 (0.02)
Sex
Boys	529 (52)	94.0 (9.7)	0.05 (0.01)	0.17 (0.02)	0.20 (0.03)
Girls	501 (48)	95.6 (10.2)	0.06 (0.01)	0.18 (0.02)	0.22 (0.03)
Race/ethnicity
White	793 (79)	99.5 (8.7)	0.05 (0.01)	0.16 (0.01)	0.19 (0.02)
Non-white	203 (21)	81.6 (9.9)	0.08 (0.01)	0.25 (0.03)	0.34 (0.03)
Missing	34	80.5 (16.0)	0.04 (0.01)	0.14 (0.05)	0.15 (0.04)
Family income (Can$)
0–59,999	369 (38)	86.1 (6.9)	0.06 (0.01)	0.19 (0.02)	0.23 (0.03)
60,000–99,999	295 (28)	91.7 (11.7)	0.05 (0.01)	0.16 (0.02)	0.18 (0.02)
≥ 100,000	337 (34)	103.0 (12.0)	0.05 (0.01)	0.17 (0.02)	0.21 (0.03)
Missing	29	167 (71.7)	0.07 (0.02)	0.21 (0.05)	0.22 (0.06)
Parental education
Less than high school diploma	170 (17)	95.3 (14.6)	0.06 (0.00)	0.19 (0.02)	0.21 (0.02)
High school diploma and above	835 (83)	97.8 (8.2)	0.05 (0.01)	0.17 (0.02)	0.21 (0.03)
Missing	25	37.0 (6.9)	0.06 (0.01)	0.19 (0.05)	0.36 (0.14)
Maternal smoking during pregnancy
No	842 (83)	95.0 (8.1)	0.06 (0.01)	0.18 (0.02)	0.22 (0.03)
Yes	154 (17)	94.2 (17.2)	0.05 (0.01)	0.16 (0.02)	0.18 (0.03)
Missing	34	91.0 (13.8)	0.05 (0.01)	0.16 (0.03)	0.21 (0.04)
Environmental tobacco smoke^*a*^
No	917 (10)	93.7 (7.3)	0.05 (0.01)	0.17 (0.01)	0.21 (0.02)
Yes	113 (90)	104.8 (16.4)	0.06 (0.01)	0.22 (0.04)	0.24 (0.05)
BMI^*b*^
Thin	809 (77)	97.2 (6.8)	0.05 (0.00)	0.17 (0.01)	0.21 (0.02)
Overweight	151 (16)	87.2 (18.2)	0.06 (0.01)	0.19 (0.04)	0.20 (0.05)
Obese	68 (7)	81.3 (12.8)	0.06 (0.01)	0.16 (0.04)	0.21 (0.05)
Missing	2	—	—	—	—
Fasting status
No (≤ 10 hr)	529 (50)	132.1 (13.5)	0.05 (0.01)	0.17 (0.01)	0.19 (0.02)
Yes (> 10 hr)	501 (50)	68.2 (5.3)	0.05 (0.01)	0.18 (0.02)	0.23 (0.03)
Blood lead concentration (μg/dL)
< 0.9	441 (42)	96.1 (8.4)	0.05 (0.01)	0.16 (0.02)	0.19 (0.03)
≥ 0.9	440 (58)	93.2 (8.1)	0.06 (0.01)	0.20 (0.02)	0.23 (0.02)
Missing	149	95.0 (12.9)	0.05 (0.01)	0.18 (0.03)	0.21 (0.04)
Pesticides used indoors^*c*^
No	993 (96)	93.5 (7.1)	0.05 (0.01)	0.17 (0.01)	0.20 (0.02)
Yes	36 (4)	129.8 (31.4)	0.13 (0.02)	0.47 (0.11)	0.61 (0.12)
Missing	1	—	—	—	—
Pesticides used outdoors^*d*^
No	935 (93)	99.1 (6.8)	0.05 (0.01)	0.17 (0.02)	0.21 (0.03)
Yes	79 (7)	83.7 (24.4)	0.06 (0.01)	0.19 (0.04)	0.26 (0.05)
Missing^*e*^	16	50.0 (17.3)	0.05 (0.03)	0.17 (0.11)	0.16 (0.11)
Pesticides to treat pets/head lice^*f*^
No	982 (96)	93.1 (7.3)	0.05 (0.01)	0.17 (0.01)	0.20 (0.02)
Yes	47 (4)	139.6 (34.2)	0.12 (0.04)	0.42 (0.14)	0.46 (0.14)
Missing	1	—	—	—	—
Any use of pesticides
No	879 (86)	93.0 (6.8)	0.05 (0.01)	0.16 (0.02)	0.19 (0.02)
Yes	151 (14)	106.0 (16.6)	0.08 (0.01)	0.29 (0.05)	0.36 (0.06)
Total	1,030 (100)	94.7 (7.2)	0.05 (0.01)	0.18 (0.01)	0.21 (0.03)
Abbreviations: —, not calculated because of insufficient observations; GM, geometric mean; SE, standard error. ^***a***^Parent report of daily tobacco smoking inside the home. ^***b***^Using the Cole method of classification (Cole et al. 1995). ^***c***^Parent-reported pesticide use in the last month for chemicals used indoors to control roaches, ants, termites, or other insects. ^***d***^Parent-reported use in the last month of chemicals on the lawn, yard, or the surrounding fields, woods, or orchards to kill insects or weeds, or to control plant diseases. ^***e***^Coefficient of variation for those estimations exceeded 30%. ^***f***^Parent-reported pesticide use in the last month for chemicals used to control head lice on family members or fleas on pets.					

In bivariate analyses (with adjustment for creatinine concentrations), ΣDAP metabolite concentrations decreased significantly with age and were higher in white children compared with nonwhite children, in children in the second and third tertiles of family income compared with children in the 1st tertile, and in children who fasted ≤ 10 hr compared with > 10 hr (all *p* < 0.05) (data not shown). For the three pyrethroid metabolites (*cis*-DCCA, *trans*-DCCA, and 3-PBA), concentrations decreased significantly with age, and were higher in nonwhite children compared with white children, and in children whose parents reported any use of pesticides (*p* < 0.05) (data not shown). In addition, 3-PBA metabolite concentrations were significantly higher among children who fasted for > 10 hr than in other children (*p* < 0.05) (data not shown).

The SDQ total difficulties scores ranged from 0 to 30 (mean = 6.9; 95% CI: 6.5, 7.4). Sixty-nine children had a high total difficulties score, suggesting a prevalence of behavioral problems of 6.8% (95% CI: 4.2, 9.4%). In bivariate analyses, a high total difficulties score was significantly associated with being a boy (9% in boys; 4.6% in girls), low parental education (8.5% in children from parents without high school diploma; 6% in children from parents with high school diploma), higher blood lead levels (8% in children ≥ 0.9 μg/dL; 4% in children < 0.9 μg/dL), maternal smoking during pregnancy (16% in children with exposure; 5% in children without), and higher maternal age at child’s birth (all at *p* < 0.05) (data not shown).

*Association between urinary levels of pesticide metabolites and behavioral problems*. Data were missing on some covariates (Table 2), so the analytical sample for the analysis of association between urinary pesticide metabolites and behavioral problems included 779 children. None of the variables listed in Table 2 was significantly different between the whole group and the subset included in the analysis (*p* > 0.05).

ΣDAP concentration was not significantly associated with high scores for total difficulties (adjusted OR for a 10-fold increase = 0.6; 95% CI: 0.3, 1.3), or the dimension scales (Table 3). Likewise, concentrations of ΣDEAP and ΣDMAP were not associated with a high total difficulties score (adjusted ORs = 0.3; 95% CI: 0.1, 1.8, and 0.8; 95% CI: 0.4, 1.6, respectively), or the dimension scales. Associations did not differ by sex for ΣDAP, ΣDEAP, or ΣDMAP high SDQ scores (*p* for interaction > 0.1) (see Supplemental Material, Table S1).

**Table 3:** Association between levels of pyrethroid and organophosphate urinary metabolites and high scores on the SDQ (≥ 17) (OR per 10-fold increase in urinary levels); *n* = 779.

SDQ	No. of cases (%)	OR (95% CI)			
		Pyrethroid *cis*-DCCA^*a*^	Pyrethroid *trans*-DCCA^*a*^	Pyrethroid 3-PBA^*b*^	Organophosphate ∑DAP^*b*^
Total difficulties	69 (6.8)	2.0 (1.1, 3.6)*	1.6 (0.9, 3.0)	1.0 (0.5, 2.0)	0.6 (0.3, 1.3)
Conduct problems	78 (8.0)	1.1 (0.4, 2.8)	1.0 (0.4, 2.2)	1.0 (0.4, 2.1)	0.6 (0.3, 1.3)
Emotional symptoms	97 (9.1)	1.4 (0.5, 4.0)	1.5 (0.5, 4.2)	1.3 (0.5, 3.6)	1.1 (0.5, 2.2)
Hyperactivity/inattention	109 (11.1)	1.4 (0.7, 2.6)	1.4 (0.8, 2.4)	1.0 (0.5, 2.0)	0.8 (0.3, 2.0)
Peer problems	71 (7.3)	0.9 (0.6, 1.5)	0.7 (0.4, 1.3)	0.7 (0.4, 1.3)	0.8 (0.3, 2.0)
^***a***^Estimates were adjusted for sex, age, race/ethnicity, income, parental education, maternal smoking during pregnancy, birth weight, blood lead levels, and urinary creatinine. ^***b***^Estimates were adjusted for the covariates listed above, plus BMI and fasting status. *p < 0.05.					

For *cis*-DCCA and *trans*-DCCA, 10-fold increases in concentrations (corresponding approximately to shifts from the 5th to the 75th percentile) were associated with adjusted ORs for a high total difficulties score of 2.0 (95% CI: 1.1, 3.6; *p* = 0.03) and 1.6 (95% CI: 0.9, 3.0; *p* = 0.12), respectively (Table 3). The association between *cis*-DCCA and high scores for total difficulties differed by sex (*p* for interaction = 0.1). There were 48 boys and 21 girls with high total difficulties scores. The association estimate was stronger for girls (adjusted OR = 3.3; 95% CI: 1.2, 8.9) than for boys (adjusted OR = 1.5; 95% CI: 0.7, 3.0) (see Supplemental Material, Table S1). We found no significant association between *cis*-DCCA and *trans*-DCCA and high scores on the dimension scales.

We observed no association between 3-PBA concentrations and high scores for total difficulties or the dimension scales (Table 3). However, there was an interaction between 3-PBA and sex for the association with high scores on the conduct problems dimension scale (*p* for interaction = 0.11). The coefficient of association was larger for girls (adjusted OR = 2.8; 95% CI: 0.7, 11.5) than for boys (adjusted OR = 0.4; 95% CI: 0.1, 2.0), but did not reach the significance level (see Supplemental Material, Table S1).

Using pesticides indoors or outdoors or to treat pets or head lice in the previous month was not significantly associated with high scores for total difficulties (see Supplemental Material, Table S2). Living in a household where pesticides were used to treat pets or head lice was significantly associated with a high score on the dimension scale for emotional symptoms (adjusted OR = 3.8; 95% CI: 1.6, 9.1). In addition, the use of pesticides for any purpose in the previous month was positively associated with high scores on the dimension scale for conduct problems (adjusted OR = 2.4; 95% CI: 0.8, 7.1; *p* = 0.10) and emotional symptoms (adjusted OR = 2.8; 95% CI: 0.8, 9.3; *p* = 0.09), although associations were not statistically significant. ORs based on unweighted analyses were similar, with significant associations of high scores on emotional symptoms with use of pesticides for pets/head lice, and for any use of pesticides (either indoor, outdoor, or pets/head lice), in the previous month (adjusted OR = 3.8; 95% CI: 1.5, 9.5 and adjusted OR = 2.7; 95% CI: 1.5, 5.1, respectively). In addition, indoor use of pesticides in the previous month was significantly associated with elevated scores on conduct problems (adjusted OR = 3.2; 95% CI: 1.0, 10.5) (see Supplemental Material, Table S2).

*Sensitivity analyses.* Analyses using creatinine-standardized metabolite concentrations (see Supplemental Material, Table S3) yielded results that were comparable with the main analysis (Table 3). Similarly, analyses without adjustment for blood lead were very similar to results of the main analysis (see Supplemental Material, Table S4). Unweighted analyses (not taking into account the survey design) also produced results similar to the main findings, with adjusted ORs for high scores for total difficulties and *cis*-DCCA and *trans*-DCCA of 2.1 (95% CI: 1.1, 4.0) and 1.8 (95% CI: 0.9, 3.4), respectively (see Supplemental Material, Table S5). In addition, the unweighted analysis resulted in a significant association between 3-PBA and high scores for conduct problems for girls (adjusted OR = 2.2; 95% CI: 1.0, 4.9), whereas the association estimate remained nonsignificant for boys (adjusted OR = 0.6; 95% CI: 0.3, 1.4) (results not shown).

Finally, blood lead levels and maternal smoking during pregnancy were significantly associated with high scores for total difficulties in all our models. For example, when adjusted for *cis*-DCCA, in addition to the other covariates, the adjusted OR for a 10-fold increase in blood lead levels was 6.6 (95% CI: 1.0, 48.3), and the corresponding OR for maternal smoking during pregnancy was 3.9 (95% CI: 1.0, 15.7) (see Supplemental Material, Table S6). Blood lead levels and maternal smoking during pregnancy also were significantly associated with high scores on conduct problems and hyperactivity/inattention dimension scales, and ORs were close to significant (*p* < 0.1) for high scores on the peer problems dimension scale.

## Discussion

The use of pyrethroids has dramatically increased over recent years, often in replacement of organophosphate pesticides that are being phased out because of concerns for human health. However, there is little information on potential health effects of environmental exposures to pyrethroid insectides. In the present study we have reported on the association between exposure to pyrethroid and organophosphate pesticides, assessed by urinary metabolites, and high levels of parent-reported behavioral problems in a sample of children from the general Canadian population. No significant positive associations were observed between organophosphate metabolites and high SDQ scores. However, a 10-fold increase in urinary levels of the pyrethroid metabolite *cis*-DCCA, which corresponds to the difference between the 75th and 5th percentiles, was associated with a doubling in the odds of having a high level of parent-reported behavioral problems. *Cis-* and *trans*-DCCA are specific metabolites of permethrin, cypermethrin, and cyfluthrin. In addition, we found some indication that domestic pesticide use might be associated with high scores for conduct problems and emotional symptoms. Because the survey is nationally representative, the findings should be generalizable to the entire population of Canadian children. This is the first study to suggest that exposure to certain pyrethroids might be associated with behavioral problems in children. These findings need to be confirmed in other populations before we can conclude that pyrethroids have detrimental effects on neurodevelopment.

Several mechanisms could underlie the association between pyrethroid insecticides and behavioral problems in children. One of the most important targets of pyrethroids is sodium channels. Pyrethroids slow the activation and inactivation of the voltage-gated sodium channels, allowing more sodium ions to cross and depolarize the neuronal membrane (Shafer et al. 2005). This increase in sodium influx could affect neuronal synaptic plasticity through modulation of the brain-derived neurotrophic factor (Imamura et al. 2006). In addition, experimental studies have reported that exposure to pyrethroid insecticides induced alterations in dopamine transporter function (Elwan et al. 2006) and influenced brain microanatomy and cholinergic/dopaminergic neurochemistry in mice (Tayebati et al. 2009).

The association between *cis*-DCCA and a high total difficulties score was stronger among girls than boys. Likewise, the association between 3-PBA and a high score for conduct disorder differed by sex, although the odds were not statistically significant for either boys or girls. The association estimates from interaction analyses were very imprecise because of the small number of cases with high scores in girls and the limited number of degrees of freedom in cycle 1 of the CHMS. Given that these findings are exploratory, we feel it is not appropriate to speculate on an explanation for this sex difference.

We did not observe an association between organophosphate metabolites in urine and high scores for hyperactivity/inattention. In a previous study among American children, we reported that urinary DAP metabolites were associated with increased odds of ADHD (Bouchard et al. 2010). This previous study classified ADHD diagnostic status based on the criteria of the *Diagnostic and Statistical Manual of Mental Disorders–Fourth Edition* (DSM-IV; American Psychiatric Association 2000). In contrast, the SDQ provides a more general assessment of mental health status, and the dimension scales might not be sufficiently sensitive to specific behavioral problems because each dimension scale is based on only five items rated on a three-level scale. Interestingly, DAP levels were higher in the present study of Canadian children [geometric mean (GM) = 94.7 nmol/L] than in the sample of American children (GM = 68 nmol/L) in the study by Bouchard et al. (2010).

Children with higher blood lead levels had significantly elevated odds for high scores on total difficulties in our study population, consistent with the well-documented effect of lead on maladaptive behaviors (for a review, see Bellinger 2008). Likewise, we observed the previously reported (McCrory and Layte 2012) association between *in utero* tobacco smoke exposure and increased levels of behavioral problems.

An important limitation of the present study is the assessment of exposure to rapidly metabolized pesticides through measurement of metabolites in only one spot urine sample. Serial measurements would have provided a better assessment of typical exposure levels. In children 3–6 years of age, a single spot sample was found to predict high (top 20%) and elevated (top 40%) DAP excretion averaged over 7 consecutive days with moderate sensitivity (*r* ≈ 0.5 and ≈ 0.7, respectively) and relatively high specificity (*r* ≈ 0.9 and ≈ 0.8, respectively) (Bradman et al. 2013). Another issue is that a fraction of urinary metabolites might reflect direct exposure through ingestion of preformed metabolites from food and other sources, rather than ingestion of the toxic parent compound. For instance, significant amounts of DAP and pyrethroid metabolites have been found in fruit juices and vegetables (Zhang et al. 2008). Whether this also applies to pyrethroid pesticides is unclear. Some degree of exposure misclassification certainly occurred on the basis of measurement of urinary metabolites, but it should be nondifferential and would tend to bias effect estimates toward the null in most instances (Jurek et al. 2005). Finally, another limitation is the small number of cases of high SDQ scores, especially when examining effect modification by sex as well as for the associations with pesticide use, resulting in imprecise association estimates.

The inferences that can be drawn from the present findings are limited by its cross-sectional design. For instance, we cannot exclude the possibility that children with behavioral problems might increase their exposure to pesticides. The findings could also be attributable to uncontrolled confounding, although we adjusted for proxy measures of several potentially important confounders, including socioeconomic and early-life factors, and blood lead levels and maternal smoking during pregnancy. Our findings were also robust when we did not account for sample weighting and survey design. Another limitation is that we did not correct for multiple comparisons, but we think this was appropriate given that there are remarkably few studies on the potential risks associated with exposure to pyrethroid pesticides. Finally, we were not able to address the question of prenatal exposure to these compounds. This important question should be addressed in further investigations.

Exposure to pyrethroids was very common in our sample. Indeed, *cis*-DCCA and 3-PBA were detected in > 97% of children’s urine samples despite the fact that pyrethroids are cleared from the body in just a few days. This suggests that exposure events are common. Questionnaire data provide some clues on the sources of exposure. For instance, 8% of children included in our study lived in a house where pesticides had been used to control pests in the house, or fleas on pets or head lice during the previous month. In addition, 7% lived in a household where chemicals had been used outdoors to control pests or weeds in the previous month. In the present study, children who lived in a family reporting any use of pesticides in the preceding month (indoors or outdoors or to treat pets and head lice) had higher levels of urinary pyrethroid metabolites than those who reported not using them. Although pyrethroids are assumed to degrade quickly by hydrolysis and photolysis, these processes might be considerably slowed indoors, thus leaving pesticide residues to linger and accumulate. In a survey conducted in 2005–2006 on homes representative of the United States, residues of permethrin were found in 89% of homes (Stout et al. 2009).

## Conclusions

Recent studies have provided compelling evidence that organophosphate pesticides have adverse effects on children’s development, and their use is decreasing. In contrast, the use of pyrethroids is increasing, but to date there is little information on potential health effects in children. The present study suggests that exposure to certain pyrethroids, at levels common in Canadian children, may be associated with behavioral problems. It is also worth mentioning that prenatal exposure to these chemicals is very poorly documented. These findings should be verified in future investigations with a longitudinal design and a larger study population.

## Supplemental Material

(455 KB) PDF
