# Supplementary material for: Urinary Metabolites of Organophosphate and Pyrethroid Pesticides and Behavioral Problems in Canadian Children
Source: Environ Health Perspect. 2013 Oct 22;121(11-12):1378–84. doi: 10.1289/ehp.1306667 (PMC3855516; doi:10.1289/ehp.1306667)
Supplement: (455 KB) PDF [file ehp.1306667.s001.508.pdf]

SUPPLEMENTAL MATERIAL

Urinary Metabolites of Organophosphate and Pyrethroid Pesticides and Behavioral Problems in Canadian Children

Youssef Oulhote[1, 2] and Maryse F. Bouchard[1, 2]

[1] Department of Environmental and Occupational Health, Université de Montréal, Québec, Canada; [2]CHU Sainte-Justine, Montréal, Québec, Canada

Table of Contents

	Page
Table S1: Association between levels of pyrethroid and organophosphate metabolites (odds ratio [OR] per ten-fold increase in urinary levels) and high scores on the SDQ by sex (weighted statistics)	2
Table S2: Association between pesticide use (pesticides indoor, outdoor, and for pets/head lice, and any pesticide use) and high scores on the SDQ (weighted and unweighted statistics; *n* = 779)	3
Table S3: Association between creatinine standardized levels of pyrethroid and organophosphate metabolites (odds ratio [OR] per ten-fold increase in urinary levels) and high scores on the SDQ (weighted statistics; *n* = 779)	4
Table S4: Association between levels of pyrethroid and organophosphate metabolites (odds ratio [OR] per ten-fold increase in urinary levels) and high scores on the SDQ without adjustment for blood lead levels (weighted statistics)	5
Table S5: Association between levels of pyrethroid and organophosphate metabolites (odds ratio [OR] per ten-fold increase in urinary levels) and high scores on the SDQ (unweighted statistics; *n* = 779)	6
Table S6: Association between blood lead levels (odds ratio [OR] per ten-fold increase) and maternal smoking during pregnancy, and high scores on the SDQ (weighted statistics; *n* = 779)	7

Supplemental Material, Table S1. Association between levels of pyrethroid and organophosphate metabolites (odds ratio [OR] per ten-fold increase in urinary levels) and high scores on the SDQ by sex (weighted statistics; *n* = 779).

		OR (95 % CI); *p*-value			
**SDQ**	**Number of cases**	**Pyrethoid *cis*-DCCA[a]**	**Pyrethoid trans-DCCA[a]**	**Pyrethoid 3-PBA[b]**	**Organophosphate ∑DAP[b]**
Total difficulties					
p-interaction		0.10	0.46	0.15	0.97
Boys	48	1.5 (0.7, 3.0); 0.26	1.4 (0.3, 2.1); 0.34	0.6 (0.3, 4.8); 0.30	0.5 (0.2, 1.8); 0.27
Girls	21	3.3 (1.2, 8.9); 0.03	2.1 (0.6, 7.0); 0.21	1.9 (0.5, 6.6); 0.29	0.6 (0.3, 1.6); 0.23
Conduct problems					
p-interaction		0.18	0.28	0.11	0.58
Boys	53	0.7 (0.2, 1.8); 0.39	0.7 (0.4, 2.2); 0.42	0.4 (0.1, 2.0); 0.15	0.7 (0.3, 1.7); 0.41
Girls	25	2.1 (0.4, 12.1); 0.36	1.6 (0.4, 6.9); 0.46	2.8 (0.7, 11.5); 0.26	0.4 (0.1, 2.0); 0.26
Emotional symptoms					
p-interaction		0.81	0.89	0.84	0.96
Boys	47	1.6 (0.5, 4.8); 0.39	1.5 (0.4, 5.4); 0.46	1.3 (0.3, 5.2); 0.71	1.0 (0.4, 3.0); 0.93
Girls	50	1.3 (0.3, 5.9); 0.69	1.4 (0.4, 5.4); 0.59	1.5 (0.5, 4.7); 0.49	1.0 (0.5, 2.0); 0.96
Hyperactivity/inattention					
p-interaction		1.0	0.90	0.60	0.21
Boys	76	1.4 (0.6, 2.9); 0.40	1.4 (0.7, 2.6); 0.27	1.0 (0.4, 2.2); 0.95	0.9 (0.4, 2.4); 0.85
Girls	33	1.4 (0.6, 3.3); 0.46	1.5 (0.7, 3.1); 0.31	1.2 (0.5, 3.1); 0.61	0.4 (0.1, 1.8); 0.21
Peer problems					
p-interaction		0.13	0.20	0.29	0.75
Boys	43	0.6 (0.3, 1.2); 0.15	0.5 (0.2, 1.2); 0.12	0.5 (0.2, 1.3); 0.13	0.8 (0.3, 2.3); 0.65
Girls	28	1.6 (0.6, 4.3); 0.33	1.2 (0.5, 3.1); 0.67	1.1 (0.4, 3.3); 0.80	0.6 (0.2, 2.7); 0.50

[a]Estimates were adjusted for age, race/ethnicity, income, parental education, blood lead levels, maternal smoking during pregnancy, and birth weight. [b]Estimates were adjusted for same covariates, BMI, and fasting status.

Supplemental Material, Table S2. Association between pesticide use (pesticides indoor, outdoor, and for pets/head lice, and any pesticide use) and high scores on the SDQ (weighted and unweighted statistics; *n* = 779).

	**Pesticides indoor**		**Pesticides outdoor**		**Pesticides for pets/head lice**		**Any pesticide use**	
	**N cases exposed**	**OR (95% CI)**	**N cases exposed**	**OR (95% CI)**	**N cases exposed**	**OR (95% CI)**	**N cases exposed**	**OR (95% CI)**
Weighted analysis								
Total difficulties	n/r	1.6 (0.3, 7.7)	n/r	2.1 (0.4, 10.2)	n/r	2.7 (0.6, 11.4)	n/r	2.1 (0.6, 7.4)
Conduct problems	n/r	2.7 (0.7, 10.8)	n/r	2.7 (0.5, 13.2)	n/r	2.0 (0.5, 7.7)	n/r	2.4 (0.8, 7.1)*
Emotional symptoms	n/r	3.9 (0.5, 28.6)	n/r	1.5 (0.3, 8.5)	n/r	3.8 (1.6, 9.1)**	n/r	2.8 (0.8, 9.3)*
Hyperactivity/inattention	n/r	0.9 (0.3, 2.8)	n/r	1.5 (0.3, 6.9)	n/r	1.7 (0.6, 5.1)	n/r	1.4 (0.5, 3.6)
Peer problems	n/r	n/c	n/r	2.0 (0.5, 8.5)	n/r	0.93 (0.2, 4.2)	n/r	1.4 (0.4, 4.5)
Unweighted analysis								
Total difficulties	5	2.7 (0.5, 13.2)	8	1.1 (0.4, 3.6)	4	2.1 (0.6, 8.0)	15	1.5 (0.6, 3.6)
Conduct problems	6	3.2 (1.0, 10.5)**	10	1.8 (0.7, 4.4)	5	1.4 (0.4, 5.1)	18	1.7 (0.8, 3.5)
Emotional symptoms	4	1.9 (0.5, 7.0)	15	2.1 (0.9, 4.7)*	8	3.8 (1.5, 9.5)**	25	2.7 (1.5, 5.1)**
Hyperactivity/inattention	3	1.1 (0.2, 5.3)	9	0.90 (0.4, 2.3)	7	1.5 (0.5, 4.8)	17	1.0 (0.5, 2.1)
Peer problems	0	n/c	6	1.3 (0.5, 3.5)	4	1.0 (0.2, 4.4)	13	1.1 (0.5, 2.5)

n/r, not reported because of the large uncertainties in estimations at the population level. n/c, not calculated because there was no exposed child with high scores on peer problems subscale.

All estimates were adjusted for sex, age, race/ethnicity, income, parental education, blood lead level, maternal smoking during pregnancy, birth weight, and urinary creatinine.

Supplemental Material, Table S3. Association between creatinine standardized levels of pyrethroid and organophosphate metabolites (odds ratio [OR] per ten-fold increase in urinary levels) and high scores on the SDQ (weighted statistics; *n* = 779).

	OR (95 % CI)			
**SDQ**	**Pyrethoid *cis*-DCCA[a]**	**Pyrethoid *trans*-DCCA[a]**	**Pyrethoid 3-PBA[b]**	**Organophosphate ∑DAP[b]**
Total difficulties	1.9 (1.0, 3.5)**	1.6 (0.8, 2.9)	1.0 (0.5, 1.9)	0.6 (0.3, 1.3)
Conduct problems	1.1 (0.4, 2.7)	1.0 (0.4, 2.2)	1.0 (0.4, 2.1)	0.5 (0.3, 1.3)
Emotional symptoms	1.4 (0.5, 3.7)	1.4 (0.5, 4.0)	1.3 (0.5, 3.5)	1.0 (0.5, 2.1)
Hyperactivity/inattention	1.5 (0.8, 2.8)	1.5 (0.9, 2.5)	1.0 (0.5, 2.1)	0.9 (0.3, 2.2)
Peer problems	0.9 (0.6, 1.5)	0.7 (0.4, 1.3)	0.7 (0.4, 1.3)	0.8 (0.3, 2.1)

[a]Estimates were adjusted for sex, age, race/ethnicity, income, parental education, blood lead levels, maternal smoking during pregnancy, and birth weight. [b]Estimates were adjusted for same covariates, BMI, and fasting status. *$p < 0.1$; **$p < 0.05$.

Supplemental Material, Table S4. Association between levels of pyrethroid and organophosphate metabolites (odds ratio [OR] per ten-fold increase in urinary levels) and high scores on the SDQ without adjustment for blood lead levels (weighted statistics).

	OR (95 % CI)			
**SDQ**	**Pyrethoid *cis*-DCCA**[a]	**Pyrethoid *trans*-DCCA**[a]	**Pyrethoid 3-PBA**[b]	**Organophosphate ∑DAP**[b]
Analysis on larger sample (*n* = 905)				
Conduct problems	1.4 (0.7 - 2.9)	1.5 (0.8 - 2.7)	1.3 (0.8 - 2.1)	0.7 (0.4 - 1.2)
Emotional symptoms	1.3 (0.5 - 3.1)	1.2 (0.5 - 2.9)	1.2 (0.5 - 2.6)	0.7 (0.4 - 1.3)
Hyperactivity/inattention	1.2 (0.7 - 2.0)	1.2 (0.7 - 2.2)	1.0 (0.6 - 1.6)	0.7 (0.3 - 1.4)
Peer problems	0.8 (0.5 - 1.4)	0.8 (0.4 - 1.3)	0.7 (0.4 - 1.2)	0.7 (0.3 - 1.5)
Analysis on smaller sample included in main analysis (*n* = 779)				
Total difficulties	2.1 (1.2, 3.8)**	1.8 (1.0, 3.3)*	1.1 (0.6, 2.1)	0.6 (0.2, 1.4)
Conduct problems	1.2 (0.5, 2.8)	1.1 (0.6, 2.3)	1.1 (0.5, 2.3)	0.6 (0.3, 1.3)
Emotional symptoms	1.5 (0.6, 3.8)	1.5 (0.6, 3.9)	1.4 (0.5, 3.5)	1.1 (0.5, 2.2)
Hyperactivity/inattention	1.5 (0.9, 2.7)	1.6 (0.9, 2.6)*	1.1 (0.6, 2.2)	0.8 (0.3, 2.1)
Peer problems	1.0 (0.6, 1.6)	0.8 (0.5, 1.4)	0.8 (0.5, 1.4)	0.8 (0.3, 2.0)

[a]Estimates were adjusted for sex, age, race/ethnicity, income, parental education, maternal smoking during pregnancy, and birth weight. [b]Estimates were adjusted for same covariates, BMI, and fasting status. *$p < 0.1$; **$p < 0.05$.

Supplemental Material, Table S5. Association between levels of pyrethroid and organophosphate metabolites (odds ratio [OR] per ten-fold increase in urinary levels) and high scores on the SDQ (unweighted statistics; *n* = 779).

	OR (95 % CI)			
**SDQ**	**Pyrethoid *cis*-DCCA[a]**	**Pyrethoid *trans*-DCCA[a]**	**Pyrethoid 3-PBA[b]**	**Organophosphate ∑DAP [b]**
Model with adjustment for blood lead levels				
Total difficulties	2.1 (1.1, 4.0)**	1.8 (0.9, 3.4)*	1.0 (0.5, 2.1)	0.6 (0.3, 1.3)
Conduct problems	1.1 (0.6, 1.9)	1.0 (0.6, 1.9)	1.1 (0.6, 2.0)	0.6 (0.3, 1.3)
Emotional symptoms	1.2 (0.7, 2.1)	1.2 (0.7, 2.1)	1.3 (0.7, 2.2)	1.1 (0.5, 2.2)
Hyperactivity/inattention	1.2 (0.7, 2.1)	1.3 (0.8, 2.2)	0.9 (0.5, 1.6)	0.8 (0.3, 2.0)
Peer problems	1.2 (0.6, 2.3)	1.0 (0.5, 1.8)	0.9 (0.5, 1.7)	0.8 (0.3, 2.0)
Model without adjustment for blood lead levels				
Total difficulties	2.1 (1.1, 4.1)**	1.9 (1.0, 3.6)*	1.1 (0.5, 2.2)	0.7 (0.4, 1.5)
Conduct problems	1.1 (0.6, 2.0)	1.1 (0.6, 2.0)	1.2 (0.7, 2.1)	0.9 (0.5, 1.7)
Emotional symptoms	1.2 (0.7, 2.1)	1.2 (0.7, 2.1)	1.3 (0.7, 2.2)	1.0 (0.5, 1.8)
Hyperactivity/inattention	1.3 (0.8, 2.2)	1.4 (0.9, 2.3)*	1.0 (0.6, 1.7)	0.8 (0.4, 1.3)
Peer problems	1.3 (0.7, 2.3)	1.0 (0.5, 1.9)	0.9 (0.5, 1.7)	0.8 (0.4, 1.6)

[a]Estimates were adjusted for sex, age, race/ethnicity, income, parental education, maternal smoking during pregnancy, birth weight, and urinary creatinine. [b]Estimates were adjusted for same covariates as above, BMI, and fasting status. **p* < 0.1; ***p* < 0.05.

Supplemental Material, Table S6. Association between blood lead levels (odds ratio [OR] per ten-fold increase) and maternal smoking during pregnancy, and high scores on the SDQ (weighted statistics; n = 779).

	OR (95% CI)	
**SDQ**	**Blood lead level** [a]	**Maternal smoking during pregnancy** [a]
Total difficulties	6.6 (1.0, 48.3)**	3.9 (1.0, 15.7)**
Conduct problems	8.6 (2.2, 41.7)**	3.5 (1.1, 11.6)**
Emotional symptoms	1.6 (0.2, 25.9)	1.1 (0.1, 9.3)
Hyperactivity/inattention	7.5 (1.7, 33.5)**	3.9 (2.2, 6.7)**
Peer problems	6.6 (0.9, 56.7)*	3.0 (0.9, 13.5)*

[a]The model included *cis*-DCCA, sex, age, race/ethnicity, income, blood lead levels, parental education, maternal smoking during pregnancy, birth weight, and urinary creatinine. *$p < 0.1$; **$p < 0.05$.
